# Binding to histo-blood group antigen-expressing bacteria protects human norovirus from acute heat stress

**DOI:** 10.3389/fmicb.2015.00659

**Published:** 2015-07-01

**Authors:** Dan Li, Adrien Breiman, Jacques le Pendu, Mieke Uyttendaele

**Affiliations:** ^1^Laboratory of Food Microbiology and Food Preservation, Faculty of Bioscience Engineering, Ghent UniversityGhent, Belgium; ^2^INSERM, UMR 892Nantes, France; ^3^CNRS, UMR 6299Nantes, France; ^4^Faculty of Medicine, University of NantesNantes, France; ^5^Nantes University HospitalNantes, France

**Keywords:** norovirus, histo-blood group antigens (HBGAs), bacteria, heat, protection

## Abstract

This study aims to investigate if histo-blood group antigen (HBGA) expressing bacteria have any protective role on human norovirus (NoV) from acute heat stress. Eleven bacterial strains were included, belonging to *Escherichia coli, Enterobacter cloacae, Enterobacter aerogenes, Clostridium difficile, Bifidobacterium adolescentis*, and *B. longum.* HBGA expression of the bacteria as well as binding of human NoV virus-like particles (VLPs, GI.1, and GII.4 strains) to the bacteria were detected by flow cytometry. NoV VLPs pre-incubated with HBGA expressing or non-HBGA expressing bacteria were heated and detected by both direct ELISA and porcine gastric mucin-binding assay. The NoV-binding abilities of the bacteria correlated well with their HBGA expression profiles. Two HBGA expressing *E. coli* (LMG8223 and LFMFP861, both GI.1 and GII.4 binders) and one non-HBGA expressing *E. coli* (ATCC8739, neither GI.1 nor GII.4 binder) were selected for the heat treatment test with NoV VLPs. Compared with the same cell numbers of non-HBGA expressing *E. coli*, the presence of HBGA-expressing *E. coli* could always maintain higher antigen integrity, as well as mucin-binding ability of NoV VLPs of both GI.1 and GII.4 after heat-treatment at 90°C for 2 min. These results indicate that HBGA-expressing bacteria may protect NoVs during the food processing treatments, thereby facilitating their transmission.

## Introduction

Noroviruses (NoVs), one genera of the *Caliciviridae* family, were reported as the cause of between 73% to greater than 95% of global epidemic non-bacterial gastroenteritis outbreaks and approximately half of all gastroenteritis outbreaks (Atmar and Estes, 2006). NoVs have a particular set of characteristics compared to other bacterial and viral pathogens that enable them to spread easily during (food borne) outbreaks and hence contribute to the global presence of this viral pathogen (Patel et al., 2009). It has been demonstrated that when reaching adulthood nearly all adults have been exposed to one or more NoVs (Donaldson et al., 2010). Although NoV infections are generally mild, it may require hospital care and can be associated with mortality in elderly, chronically ill or immune-compromised patients. Taken into consideration of their widespreadness, NoVs are causing heavy disease burdens associated with large economic losses (Van Beek et al., 2013).

Human NoVs have been very difficult to study since being discovered in 1972, as the cellular tropism is unknown, they cannot be grown in culture, and there is no robust small-animal model. Numerous efforts have been made to search for an *in vitro* infection model for human NoVs (Duizer et al., 2004; Papafragkou et al., 2013) until very recently a breakthrough has been reported (Jones et al., 2014). Human NoVs have been observed to bind to histo-blood group antigens (HBGAs) in a strain-specific manner both *in vitro* and *in vivo* (Tan and Jiang, 2010; Ruvoën-Clouet et al., 2013). On the other hand, an individual’s susceptibility to NoVs is correlated with his HBGA profile *in vivo* (Tan and Jiang, 2010; Ruvoën-Clouet et al., 2013). As a number of gram-negative bacteria show blood group expression (Springer et al., 1961), it has been recently confirmed that NoVs could bind to an enteric bacterium strain (SENG-6) closely related to *Enterobacter cloacae* bearing HBGA-like molecules located in their extracellular polymeric substances (EPS; Miura et al., 2013). In the study of Jones et al. (2014) B cells were identified as a cellular target of NoVs, and the presence of HBGA expressing enteric bacteria were required for human NoV infection of B cells. Based on these facts, we found it of interest to investigate if the HBGA-expressing bacteria have any protective role on NoV.

This study aimed at identifying a few HBGA-expressing bacteria, to confirm the specific binding of human NoVs to the HBGA-expressing bacteria, and to investigate the protective effect of these bacteria on NoVs toward heat treatment. *Escherichia coli, Enterobacter cloacae, Enterobacter aerogenes* strains were included as they are closely related with the reported HBGA-expressing strains (Springer et al., 1961; Miura et al., 2013). Among different treatments of NoV, the use of high temperature has been proven to be the most direct and effective strategy, which is also most widely used in food processing industries for food safety control and preservation (Rahman, 2007). As reviewed by Hirneisen et al. (2010), the mechanism of heat inactivation of viruses is believed to be due to changes in the capsid of the virus particle. Therefore, this study investigated the protective effect of bacteria that express HBGA antigens for NoV during heat stress.

## Materials and Methods

### Bacteria and Virus-Like Particles (VLPs)

Eleven bacterial strains listed in **Table 1** were selected from ATCC, Belgian Coordinated Collection of Microorganisms (BCCM/LMG), or self-isolated strains of the Laboratory of Food Microbiology and Food Preservation (LFMFP), Ghent University. All eleven bacteria were cultured in tryptone soya broth (TSB, Oxoid, Thermo) at 37°C. For *Clostridium difficile, Bifidobacterium adolescentis*, and *B. longum*, the anaerobic atmosphere was generated with the use of ANAEROGEN^TM^ COMPACT (Oxoid, Thermo).

**Table 1:** Histo-blood group antigen (HBGA) expression of the bacteria and binding of human norovirus (NoV) virus-like particles (VLPs) to the bacteria.

Bacteria	Strain	Biological origin	HBGA expression									VLP binding	
			anti-A 21	anti-B 49	anti-H Mbr1	anti-H 53	anti-H 19-0Le	anti-Le^a^ 7-Le	anti-Le^b^ 2-25-Le	anti-Le^x^ 3E1	anti-Le^y^ 12-4Le	GI.1	GII.4
*Escherichia coli*	LMG8223	Clinical isolate	+++	++	+	++	+	++	+	+	++	+	+++
*E. coli*	LFMFP861	Thick whey products	-	+++	-	-	-	++	-	-	-	++	+++
*E. coli*	LFMFP289	Pork	-	+	-	-	-	-	+	-	-	++	+
*E. coli*	LFMFP853	Lettuce	-	+	-	-	-	+	-	-	-	-	-
*E. coli*	LFMFP134	Carcass	-	-	-	-	-	+	-	-	-	-	-
*E. coli*	ATCC8739	Feces	-	-	-	-	-	-	-	-	-	-	-
*Enterobacter aerogenes*	LMG2094	Sputum	-	-	-	-	-	+	+	-	+	+	-
*Enterobacter cloacae*	LMG2783	Cerebrospinal fluid	-	-	-	-	-	+	+	-	-	-	-
*Clostridium difficile*	LMG21717	Unspecified	-	-	-	-	-	+	-	-	-	+	-
*Bifidobacterium adolescentis*	LFMFP422	Unspecified	-	-	-	-	-	-	-	-	-	-	-
*B. longum*	LFMFP425	Unspecified	-	-	-	-	-	-	-	-	-	-	-

*The percentage of positively stained cells determined by flow cytometry analysis were grouped as in the following. <1%: -; 1%∼5%: +; 5%∼10%: ++; > 10%: +++.*

A series of anti-HBGAs antibodies was used. Anti-A antibodies #3, #5, #7, #21 and anti-B antibodies #38, #39, #40, #43, #49 have been described previously (Le Pendu et al., 1997). The anti-H type 3/4 Mbr1 was obtained from Enzo Life Sciences; the anti-H 19-0LE, an anti-H type 2 that shows some cross-reactivity with Le^y^, the anti-Le^a^ 7-LE, the anti-Le^b^ 2-25LE, and the anti-Le^y^ 12-4LE were obtained from Dr. J. Bara (CNRS, Villejuif, France). The anti-Le^x^ 3E1 was obtained from Dr. D. Blanchard (EFS, Nantes, France). The specificities of the monoclonal (mAb) anti-HBGAs used in the study were described in **Table 2**.

**Table 2:** Specificities of the monoclonal (mAb) anti-HBGAs used in the study.

	mAb dentification	Specificity	Dilution
Anti-A	#3	A types 3/4	1:10
	#5	A type 2	1:10
	#7	A type 5, ALe^y^ > ALe^b^	1:10
	#21	A types 1-5, ALe^b^	1:50
Anti-B	#38	B type 2, BLe^y^	1:10
	#39	B type 2	1:10
	#40	B type 2	1:10
	#43	B type 2	1:10
	#49	B types 1-5, BLe^y^	1:10
Anti-H/Lewis	Mbr1	H types 3/4	1:60
	19-OLE	H type 2 > Le^y^	1:300
	#53	H type 2	1:10
	12-4LE	Le^y^	1:300
	2-25LE	Le^b^> Le^a^	1:100
	7-LE	Le^a^	1:100
	3E1	Le^x^	1:10

Virus-Like Particles of human NoV GI.1 (Norwalk virus) and GII.4 (Dijon 1996) were generated using a previously described method (Caddy et al., 2014). Recombinant baculoviruses containing the VP1 protein from NoV GI.1 and GII.4 were generated, and VLPs were produced by infection of Hi5 insect cells. VLPs were released from the infected Hi5 cells by three rounds of freeze-thawing and then clarified by removal of cellular debris (6,000 × *g* for 30 min) and baculovirus (14,000 × *g* for 30 min). The VLPs were partially purified through a 30% (wt/vol) sucrose cushion in TNC buffer (50 mM Tris-HCl, pH 7.4, 150 mM NaCl, 10 mM CaCl_2_) containing the protease inhibitor leupeptin at 150,000 × *g* for 2 h. The pelleted VLPs were resuspended in TNC and further purified by isopynic centrifugation in cesium chloride (150,000 × *g*; 18 h). The resultant VLP bands were collected by puncture, and the solution containing VLPs was dialyzed against PBS prior to quantification by bicinchoninic acid (BCA) protein assay (Thermo Scientific) and storage at -80°C.

### Flow Cytometry Analysis

For the HBGA expression test, 6 h cultures of *E. coli, Enterobacter cloacae*, and *Enterobacter aerogenes* (plateau growth phase), and 48 h culture of *Clostridium difficile, B. adolescentis*, and *B. longum* (plateau growth phase) were collected. The concentrations of bacteria culture were normalized with TSB to an OD_570_ of 0.4 (around 10^8^ CFU/ml). In the following the bacterial pellet (100 μl per sample) were washed twice with phosphate buffered saline (PBS, pH 7.4) before being re-suspended in PBS-0.1% bovine serum albumin (BSA, 100 μl per sample) and incubated at 37°C for 1 h to block the non-specific binding. After centrifugation at 6,000 × *g* for 5 min, mouse monoclonal antibodies (**Table 2**) diluted in PBS-0.1% BSA were added to the bacteria pellets, mixed by vortex for a few seconds and incubated at 37°C for 1 h. HBGAs were detected using a series of mouse monoclonal anti-A, anti-B, anti-H and anti-Lewis antibodies that present different specificities HBGA subtypes. An irrelevant IgG3 (Sigma) was included as the negative control. After two washings by centrifugation at 6,000 × *g* for 5 min in PBS, the last incubation was performed with Alexa Fluor^®^ 488 Goat anti-mouse Fab fragment (Beckman Coulter) at dilution of 1:200 in PBS-0.1% BSA at 37°C for 1 h. After final washes (centrifugation at 6,000 × *g* for 5 min in PBS), fluorescence analysis was performed on a BD^TM^ LSR II flow cytometer (5,000 events were set-up as the gating, Becton Dickinson, Rungis, France).

The binding of human NoV VLPs to the bacteria was identified using the above protocol with a few variations. Human NoV VLP of GI.1 and GII.4 were diluted in PBS-0.1% BSA to 5 and 10 μg/ml, respectively, added to the washed and blocked bacteria pellet at 100 μl per sample, mixed and incubated at 37°C for 1 h. After washing, anti-VLP rabbit polyclonal antibodies (lp130 for GI.1 and lp132 for GII.4, self-produced antibody by INSERM) diluted in PBS-0.1% BSA at 1:200 were added, incubated and washed, followed by incubation with Dylight^TM^ 488 Goat Anti-Rabbit IgG (H + L) antibody (KPL, Gaithersburg, MD, USA) at a dilution of 1:200 in PBS-0.1% BSA. After final washes, fluorescence analysis was performed on the BD^TM^ LSR II flow cytometer (5,000 events were set-up as the gating, Becton Dickinson).

In order to control for the specificity of the anti-HBGA antibodies and of VLPs binding to bacteria, *E. coli* strains LMG8223 was treated by α-*N*-acetylgalactosaminidase (New England Biolabs) at 37°C for 1 h. Chinese hamster ovary (CHO) cells, transfected with rat α1,2- fucosyltransferase B cDNA and the rat A enzyme cDNA as described previously (Marionneau et al., 2002), were utilized as a positive control for this enzymatic treatment. *E. coli* LFMFP861 was treated by the α-galactosidase from coffee beans (Sigma) at 37°C for 1 h. The A and B antigens expression on treated and untreated cells were detected by the use of antibody #21 and #49, respectively. Additionally, *E. coli* LFMFP861 was treated by the α-galactosidase (Sigma) and an α1,2-fucosidase from *B. bifidum*, respectively, at 37°C for 1 h. The binding of human NoV VLPs GI.1 to the untreated and treated bacteria were then quantified as above.

### Fluorescence Microscopy

*Escherichia coli* LMG8223 was stained with the use of antibody #21 and an irrelevant IgG3, respectively, as described for the flow cytometry experiments. In the final incubation step, besides Alexa Fluor^®^ 488 Goat Anti-Mouse IgG (H + L) antibody (Life Technologies), the blue-fluorescent Hoescht nucleic acid stain (NucBlue Live stain, Life Technologies) was also included. The stained cells were fixed with 4% paraformaldehyde and mounted on slides with Vectashield^®^ (Vector laboratories, Burlingame, CA, USA). The sealed slides were observed under a Zeiss Axiovert 200 fluorescence microscope.

### Heat Treatment

*Escherichia coli* LMG8223, LFMFP861, and ATCC8739 (100 μl per sample) were normalized to an OD_570_ of 0.4 and washed with PBS. Human NoV VLPs diluted in PBS (GI.1 at 2.5 μg/ml and GII.4 at 5 μg/ml) were added to the bacterial pellet (100 μl per sample) and mixed well by vortexing. The mixtures (100 μl per sample in 0.5 ml thin-walled PCR tubes) were heated at 90°C for 2 min in a PCR cycler (MJ Research PTC200 Thermal Cycler) and placed on ice to cool down before further tests.

### ELISA-based Microtiter Plate Assays

For the direct ELISA, the mixtures of NoV VLPs and bacteria were diluted in carbonate buffer (CBS, 50 mM sodium carbonate, pH 9.6 at 1:10, directly coated on Nunc Maxisorp immunoplates and incubated overnight at 4°C. The plates were washed three times with 0.05% Tween 20 in PBS (PBS-T) and then blocked with 5% skim milk (BD Difco^TM^) in PBS for 1 h at 37°C. Anti-VLP rabbit polyclonal antibodies (lp130 for GI.1 and lp132 for GII.4) diluted in PBS-5% skim milk (BD Difco^TM^) at 1:1,000 were added, and another 1-h incubation at 37°C took place. After three times washing with PBS-T, the final 1-h incubation at 37°C was with horseradish peroxidase (HRP)-conjugated anti-rabbit IgG (Uptima) at a 1:4,000 dilution in 5% skim milk (BD Difco^TM^) in PBS. After three times washing with PBS-T, the enzyme signals were detected using TMB (3,3′,5,5′-tetramethylbenzidine) as the substrate (BD Bioscience, San Jose, CA, USA), and the reaction was stopped with 1 N phosphoric acid. The optical density was read at 450 nm (OD_450_). The non-coated wells were included as blank controls.

Porcine gastric mucin, which contains mixed type A, H type 1, and Lewis b HBGAs was reported to be a good candidate for binding of many human NoVs strains (Tian et al., 2008). For the porcine gastric mucin-binding assay, Nunc Maxisorp immunoplates were pre-coated with porcine gastric mucin (Sigma M1778, 2 μg/well) in CBS overnight at 4°C. The plates were washed three times with PBS-T. The wells were then blocked with 5% skim milk (BD Difco^TM^) in PBS for 1 h at 37°C before the mixtures of NoV VLPs and bacteria (untreated and treated) were added to each well and incubated for 1 h at 37°C. After three times washing with PBS-T, the following procedures (incubation with antibodies and HRP activity measurement) were performed as for the direct ELISA described above, except the enzyme signals were detected with TMB One Solution (Promega). The mucin-coated wells without the addition of VLPs were included as blank controls.

### Data Analysis

Statistical analyses were performed by one-way analysis of variance (the Tukey’s test was used as a *post hoc* test) with SPSS 22 for Windows (SPSS, Inc., Chicago, IL, USA). Significant differences were considered when *P* was <0.05.

## Results

### HBGA-Expression and Human NoV VLP-Binding to the Bacteria

**Table 1** shows the results of an initial screening of the HBGA expression and NoV VLPs binding ability of the eleven bacterial strains detected by flow cytometry analysis. The NoV-binding abilities of the bacteria correlated well with their HBGA expression profiles. Both human NoV VLPs GI.1 and GII.4 bound to *E. coli* LMG8223, which had high expression of type A antigen (42.5%) and lower expressions of type B, H, and Lewis antigens (1∼10%) and *E. coli* LFMFP861, which had high expression of type B antigen (14.3%) and lower expression of Lewis a antigen (6.2%). Bacterial strains with weak HBGA expressions (*E. coli* LFMFP289, LFMFP853, LFMFP134, *Enterobacter aerogenes* LMG2094, *Enterobacter cloacae* LMG2783, and *Clostridium difficile* LMG21717) showed weak or no NoV binding ability. Bacteria without HBGA expression (*E. coli* ATCC8739, *B. adolescentis* LFMFP422, *B. longum* LFMFP425) showed no NoV binding ability.

Followed by the initial screening as shown in **Table 1**, the HBGA expression and NoV VLPs binding for *E. coli* LMG8223 and *E. coli* LFMFP861 were further analyzed.

Firstly, the type A antigen expression on *E. coli* LMG8223, the type B antigen expression on *E. coli* LFMFP861 and the binding of NoV VLPs GI.1 and GII.4 to *E. coli* LMG8223 and *E. coli* LFMFP861 were repeated independently three times by flow cytometry analysis (**Table 3**). The qualitative positive results were confirmed repeatedly while the percentage of positively stained cells varied largely probably due to the variation of HBGA expression on bacteria cells from different batches (although the bacteria were always inoculated at the same cell numbers and cultured for the same time).

**Table 3:** The HBGA expression and binding of human NoV VLPs to the bacteria *E. coli* LMG8223 and *E. coli* LFMFP861 (percentage of positively stained cells determined by flow cytometry analysis, three independent tests).

		Average (%)	SD (±%)
*E. coli* LMG8223	Type A antigen expression	28.5	13.4
	VLP GI.1 binding	5.6	1.5
	VLP GII.4 binding	10.9	8.3
*E. coli* LFMFP861	Type B antigen expression	6.8	6.6
	VLP GI.1 binding	9.8	4.3
	VLP GII.4 binding	14.2	18.7

**FIGURE 1:**
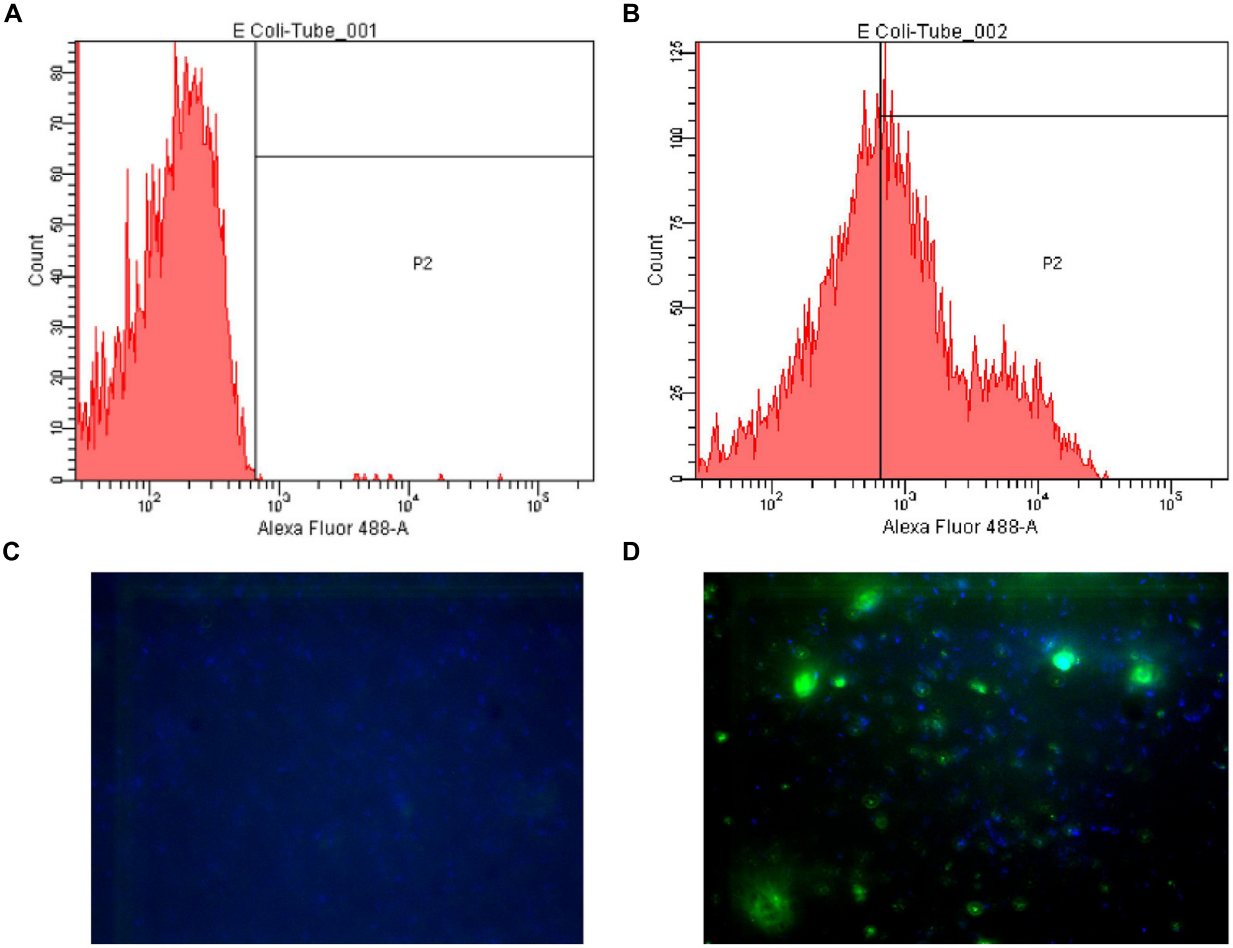
**Examples of flow cytometry and fluorescence microscopy results. (A)** Flow cytometry plot of *Escherichia coli* LMG8223 stained by an irrelevant IgG3 (negative control); **(B)** Flow cytometry plot of *E. coli* LMG8223 stained by anti-A antibody #21; **(C)** Fluorescence microscopy picture of *E. coli* LMG8223 stained with an irrelevant IgG3 (negative control); **(D)** Fluorescence microscopy picture of *E. coli* LMG8223 stained by anti-A antibody #21.

Secondly, the fluorescence microscopy results, which showed *E. coli* LMG8223 stained by anti-A antibody #21, were supplied as a visual control of the flow cytometry analysis (**Figure 1**).

Thirdly, in order to get some insight into the HBGA structures present on bacteria, we used several different anti-A, B, and H antibodies that react with more or less restricted sets of A, B, and H epitopes. Indeed these can be mono or difucosylated and vary according to the type of precursor structure (types 1–5; Marionneau et al., 2001). We observed that those antibodies reactive with either *E. coli* LMG8223 or *E. coli* LFMFP861 were those that have the broadest reactivity to A or B epitopes. Thus, *E. coli* LMG8223 showed positive reactions with anti-A antibodies #21 (42.5%) and #7 (68%), which have very strong, and broad A reactivity, but not with antibodies #3, or #5 (<1%), which have more restricted anti-A specificity (Le Pendu et al., 1997). Similarly, *E. coli* LFMFP861 showed strong positive reaction with anti-B antibody #49 (14.3%), which has a broad reactivity with a group of B-like molecules, but showed low or no reaction with antibodies #38 (3.9%), #39 (2.7%), #40 (<1%), and #43 (3.9%), known to be more restricted anti-B (Le Pendu et al., 1997). These results indicate that not all anti-HBGA recognize bacteria, even though they bind to human red cells.

Furthermore, in order to confirm specificity of the anti-A or B staining of the bacteria, we attempted to remove these epitopes using specific glycosidases. An α-*N*-acetylgalactosaminidase that specifically catalyzes the hydrolysis of α-linked *N*-acetylgalactosamine residues from the blood group A epitope and an α-galactosidase that removes the terminal galactose residue of the blood group B epitope were used. Treatment of CHO cells expressing the A antigen by the α-*N*-acetylgalactosaminidase clearly decreased the A antigen expression from 66.8% positive cells as detected by flow cytometry to 28.6%, showing efficacy of the enzyme treatment. It had a more limited effect on the A antigen expression of *E. coli* LMG8223 (From 74.8 to 64.9%), suggesting that the bacteria blood group A structures might be slightly different from mammalian structures which serve as substrates for this glycosidase. This could also explain why NoV GI.1 showed less binding to *E. coli* LMG8223 than GII.4 (**Table 1**), which has more flexible binding sites based on the observation from crystallographic studies (Prasad et al., 2014).

Treatment of *E. coli* LFMFP861 with α-galactosidase decreased the B antigen expression (from 14.3 to 3.6%) unambiguously demonstrating specificity of the anti-B staining. NoV GI.1 binding to that *E. coli* strain was quite surprising since it does not recognize either B or Lewis a blood group antigens (Huang et al., 2005) which were the only ones that we could evidence on *E. coli* LFMFP861 (**Table 1**). It was therefore hypothesized that there might be an unknown H-type structure present on *E. coli* LFMFP861, which does not react with the anti-H antibodies used in this study, but could bind to NoV GI.1. Consistent with this hypothesis, the binding of NoV GI.1 VLP to *E. coli* LFMFP861 was decreased following pre-treatment of the bacteria with an α1,2-fucosidase that removes the α1,2-linked fucose which constitutes the essential building block of H antigens (from 9.2 to 6.6%). Furthermore, NoV GI.1 binding to *E. coli* LFMFP861 was increased after a pre-treatment of the bacteria with the α-galactosidase, which likely exposed more H antigen on the bacteria (from 9.2 to 13.1%), further confirming specificity of the VLPs binding. The exact structure of the bacterial HBGAs remains to be determined. Nevertheless, an authentic B blood group tetrasaccharide from *E. coli* 086 has been previously characterized (Yi et al., 2005). Likewise, an α1,2-fucosyltransferase was isolated from *E. coli* 0127:K63 (Pettit et al., 2010). This enzyme specifically synthesized H type 3 only, which may not be an optimal substrate for the α1,2-fucosidase that we used and which is not recognized by most anti-H antibodies. The low effect of either the α-*N*-acetylgalactosaminidase or the α1,2-fucosidase on anti-A or GI.1 VLPs binding, respectively, might thus be due to differences in the precise blood group epitope and to the context of their presentation on the bacterial surface as compared to that of the cognate mammalian epitopes.

### Protection of NoV VLPs from Heat Treatment by HBGA Expressing Bacteria

**FIGURE 2:**
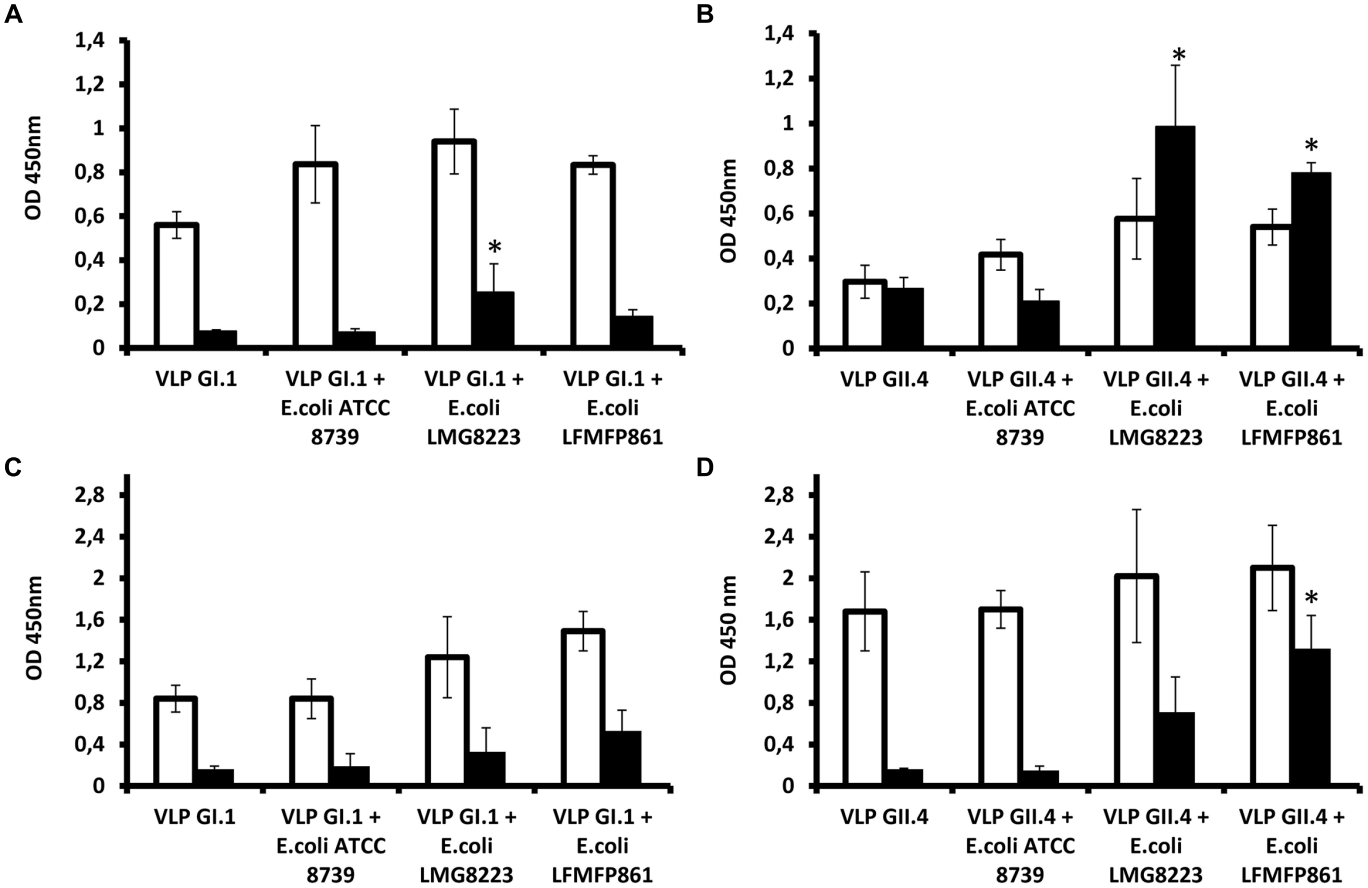
**Protection of histo-blood group antigen (HBGA) expressing bacteria on human norovirus (NoV) virus-like particles (VLPs) toward heat treatment. (A,B)** Antigenicity detection of NoV GI.1 and GII.4 in the absence of bacteria, in the presence of non-HBGA expressing *E. coli* ATCC8739 or HBGA-expressing *E. coli* LMG8223 and LFMFP861 before (white bars) or after (black bars) heat treatment at 90°C for 2 min. **(C,D)** Mucin-binding ELISA results of NoV GI.1 and GII.4 in the absence of bacteria, in the presence of non-HBGA expressing *E. coli* ATCC8739 or HBGA expressing *E. coli* LMG8223 and LFMFP861 before (white bars) or after (black bars) heat treatment at 90°C for 2 min. ^∗^Significant differences as explained in the Section “Results.” Each data point is an average of three independent tests, and each error bar represents the data range.

In order to determine if the HBGA-dependent attachment of NoVs to bacteria could play some role in the virus transmission, we tested if it could protect virions from acute heat stress as encountered during cooking. To this aim, *E. coli* LMG8223, *E. coli* LFMFP861 (both GI.1 and GII.4 binders), and *E. coli* ATCC8739 (neither GI.1 nor GII.4 binder) were used for the heat treatment test with NoV VLPs. Direct ELISA and mucin-binding ELISA were performed in order to measure the antigenicity and receptor-binding ability of NoV VLPs, respectively. Three independent tests were performed and the results (OD_450_) are shown in **Figure 2**. Blank controls as the background were with OD_450_ values around 0.1.

In the absence of bacteria or in the presence of non-HBGA expressing *E. coli* ATCC8739, detection of NoV GI.1 VLPs by a specific polyclonal antiserum was decreased to background level after 90°C treatment for 2 min. By contrast, the immunoreactivity of the VLPs remained after the heat treatment upon incubation with HBGA expressing bacteria *E. coli* LMG8223 and LFMFP861, of which VLPs with *E. coli* LMG8223 showed significantly (*p* < 0.05) higher immunoreactivity after the heat treatment than NoV GI.1 VLPs without bacteria or with *E. coli* ATCC8739 or LFMFP861 (**Figure 2A**). Likewise although not significantly, higher porcine mucin binding levels of NoV GI.1 VLPs were obtained after heat treatment upon incubation with HBGA expressing bacteria *E. coli* LMG8223 and LFMFP861 than NoV GI.1 VLPs without bacteria or with non-HBGA expressing *E. coli* ATCC8739 (**Figure 2C**).

When the same experiments were performed using NoV GII.4 VLPs, the direct ELISA results showed that GII.4 were more resistant to heat treatment than GI.1, which is contrary to previous assumptions that GI are more resistant than GII to environmental conditions and removal during the sewage treatment process (da Silva et al., 2007). Indeed, detection of GII.4 VLPs without bacteria and with non-HBGA expressing *E. coli* ATCC8739 by direct ELISA decreased after heat treatment, albeit not to background level, whilst in the presence of HBGA-expressing *E. coli* LMG8223 and *E. coli* LFMFP861, detection of GII.4 epitopes increased after heat treatment and showed significantly (*p* < 0.05) higher immunoreactivity than NoV GII.4 VLPs without bacteria or with non-HBGA expressing *E. coli* ATCC8739 (**Figure 2B**). It is likely that in the latter condition, the immunodetection of GII.4 VLPs was higher following heat treatment than in the non-heated control due to partial degradation of the VLPs that resulted in unmasking of epitopes. After heat treatment, the mucin-binding ability of GII.4 VLPs was also quite well preserved by incubation with HBGA-expressing bacteria, whilst it was entirely lost in the absence of bacteria or in the presence of HBGA non-expressing *E. coli* ATCC8739. NoV GII.4 VLPs with *E. coli* LFMFP861 showed significantly (*p* < 0.05) higher mucin-binding level than NoV GII.4 VLPs without bacteria or with *E. coli* ATCC8739 or LMG8223 (**Figure 2D**). These results indicated that the presence of HBGA-expressing bacteria could help maintain integrity of GII.4 VLPs upon heat treatment.

## Discussion

The major observation of this study is that compared with the same cell numbers of non-HBGA expressing *E. coli*, the presence of HBGA-expressing *E. coli* could maintain higher antigen integrity as well as mucin-binding ability for both NoV VLPs GI.1 and GII.4 after heat-treatment at 90°C for 2 min. In general, studies on the HBGA expression of bacteria are not abundant in literature, therefore in this study efforts were made firstly on the HBGA identification and confirmation on the selected bacterial strains before studying on the effect of the HBGA structures on viruses.

It is shown in this study that there can be large variations of HBGA expression between different bacterial strains (even closely related genetically). Moreover, the HBGA expression levels of one particular bacterial strain can vary to large extents (as shown in **Table 3**) from different cultural batches (although the bacteria were always inoculated at the same cell numbers and cultured for the same time). This is not very surprising as the HBGA-like substances can be secondary metabolites of bacteria and the expression can be regulated by some unknown factors, which still need to be studied in the future. Therefore the HBGA expression of *E. coli* LMG8223 and *E. coli* LFMFP861 were confirmed qualitatively, and the data are considered to be quantitatively comparable only for samples from the same batches (5000 cells were analyzed by flow cytometry for each sample).

Compared with previous studies (Springer et al., 1961; Miura et al., 2013), the use of multiple specific monoclonal ABH and Lewis antibodies in this study supplied more information on the variable HBGA-structures of different bacteria. In addition, the reactions of *E. coli* LMG8223 and *E. coli* LFMFP861 with several different anti-A, B, and H antibodies that react with more or less restricted sets of A, B, and H epitopes, and results from treating A or B epitopes on these two bacteria using specific glycosidases indicated that the HBGA epitopes present on bacteria may not be identical with those present on human red cells (A type 2, B type 2, or H type 2). It was previously reported that oysters, which are important vectors for human NoVs, may play a selective role in the transmission of different NoV strains via specific binding to carbohydrate ligands (Le Guyader et al., 2013). According to this study, the binding ability of different NoV strains to HBGA-expressing bacteria can be variable, indicating that the HBGA-expressing bacteria, which can spread widely in the nature (Springer et al., 1961), may contribute to the NoV strain selective transmission as well.

The reactions of human NoVs toward heat treatments have been studied by many researchers. As reviewed by Bertrand et al. (2012), these studies were conducted under different conditions with variable models and methods. Our results in this study is consistent with the report of Hirneisen and Kniel (2013) showing that heating of a NoV GII strain at 80 and 100°C for 5 min could decrease the binding of the virus to porcine gastric mucin to levels under the cut-off.

It has to be noted that the results obtained on NoV VLPs from direct ELISA and mucin-binding ELISA may not mirror authentic NoV infectivity. Nevertheless, these results do suggest that HBGA-expressing bacteria may protect NoVs during the food processing treatments and possibly in the human gastrointestinal tract before causing infection. Through this protection effect, HBGA-expressing bacteria may facilitate the virus transmission. In the future, more treatment conditions should be tested and the protective mechanisms of the HBGA-expressing bacteria on NoVs should be investigated, especially if a routine method can be established to measure infectivity of human NoVs.

## Conflict of Interest Statement

The authors declare that the research was conducted in the absence of any commercial or financial relationships that could be construed as a potential conflict of interest.
